# A Treatise on the Diseases of Children. Affections of the Chest. First Part, Pneumonia

**Published:** 1841-10

**Authors:** 


### Art. III.

1. *Maladies des Enfans. Affections de Poitrine.* Première Partie, Pneumonie. Par MM. Rilliet et Barthez.—*Paris*, 1838. 8vo, pp. 238.

*A Treatise on the Diseases of Children. Affections of the Chest.* First Part, Pneumonia. By Messrs. Rilliet and Barthez.

2. *Recherches sur la Bronchite capillaire, purulente et pseudo-membraneuse,* (*Catarrhe suffocant, Croup bronchique*), *chez les Enfants.* Par S. A. Fauvel, m.d. &c.—*Paris*, 1840. 4to, pp. 100.

*Researches on the purulent and pseudo-membranous form of Capillary Bronchitis in Children,* (*otherwise called suffocative Catarrh, or bronchial Croup.*) By S. A. Fauvel, d.m.p.—*Paris*, 1840.

3. *Ueber die acute Bronchitis der Kinder, und ihr Verhältniss zu den verwandten Krankheitsformen.* Von Dr. W. Cruse, &c.—*Königsberg*, 1839.

*On the acute Bronchitis of Children, and its Relation to similar Diseases.* By Dr. W. Cruse, &c.—*Königsberg*, 1839. 8vo, pp. 187.

4. *Ein Beitrag zur Lehre von der Bronchitis der Kinder.* Von Dr. R. Küttner, pract. Arzte in Dresden. (*Casper's Wochenschrift, Juni*, 1841.)—*Berlin.*

*An Essay on the Bronchitis of Children.* By Dr. Küttner, of Dresden. —*Berlin*, 1841.

In his treatise, " De aere, aquis et locis," the father of our art has mentioned the precautions which a practitioner of medicine should observe on settling in a foreign city. He directs him to examine the situation of the place, its climate, and the direction from which the wind ordinarily blows. The food, the dress, the habits, occupations and amusements of the inhabitants are all to be carefully noted; for, without a knowledge of these things no one can judge correctly of their diseases, nor adopt the measures most proper for their cure.

The lapse of ages has not detracted from the value of Hippocrates's advice; but our countrymen appear sometimes to have forgotten its importance, when appealing to the authority of French writers on the diseases of children. The field in which MM. Billard, Valleix, Denis, Berton, and others laboured, is one to which England, fortunately as we think, presents no parallel. No one would regard the strange and fearful forms of disease which he might observe in a city wasted by pestilence or famine, as affording a correct idea of the habitual health of the inhabitants; though to do so would be little less illogical than to apply to the diseases of children in general the results afforded by the hospitals of Paris.

There are two hospitals in Paris appropriated to children, the *Hospice des Enfans Trouvés, and the Hôpital des Enfans Malades.* The foundling hospital receives children from their birth until two years of age, and, like similar institutions in the rest of France, places them at nurse, and provides for their after support. The mortality in these institutions is frightfully great. Sixty per cent. of the foundlings throughout the whole of France die before attaining the age of one year; though the discrepancies are extreme between the reported mortality in different provinces; being, according to M. de Chateauneuf, only 15·25 per cent.

in Alsace, while in le Maine and the Bourbonnais it is 75·50, and 80·04 per cent.* The average stay of children in the Hospice at Paris does not exceed 9½ days, during which short time the mortality is 1 in 4·3, while the proportion of deaths to recoveries among the sick is nearly as four to one.†

With this high rate of mortality in the foundling hospital at Paris, let us contrast that in the lying-in hospital at Leipsig, where the children remain fifteen days, and die in the proportion of 1 to 41·13.‡ The number of cases yielding this result was indeed very limited, being only 1284, but the report of Dr. Collins gives us a still more favorable result, though deduced from 16,654 cases; 284 only of which, or 1 in 58½, died during a residence of eight, nine, or ten days in the hospital.§ So powerful an influence do warmth, pure air, and the suckling of infants by their own mothers exert in diminishing their mortality. In 1782 nearly a sixth of all children born in the Dublin lying-in hospital died within the first fortnight, and the present low rate of mortality is above all to be attributed to the excellent ventilation and scrupulous cleanliness which pervade that noble institution.

We should not, however, insist thus upon the mortality of the hospital at Paris, if the children there died of the same diseases as when placed under other circumstances; but that is not the case. When large numbers of young children are congregated together, diseases prevail among them endemically which are hardly met with elsewhere. In Paris the *endurcissement du tissu cellulaire* and *muguet* prevail in the same way as trismus did formerly in Dublin.‖ The various organs, and especially the lungs, acquire a proneness to inflammation truly surprising. In 1834, M. Savatier published a paper in La Clinique,¶ founded on the dissection of thirty-four children who formed the whole mortality under M. Baron, between September 14 and October 5, 1827. He found the lungs more or less inflamed in every instance, on the strength of which fact he asserts that pneumonia invariably complicates all diseases of new-born infants. M. Valleix embodies, in his essay on Infantile Pneumonia, the results of 114 dissections made by M. Vernois.** The cases were examined indiscriminately during the months of February, April, and March; and in 113 hepatization of the lungs was found to exist. M. Vernois' observations indeed were made during the prevalence of influenza, but the investigations of M. Savatier, which were not influenced

* History of public charity in France, by D. Johnstone, M.D., Edinb. 1829. 8vo. p. 367. It appears from the Registrar-General's Report, that the mortality of children under one year old, throughout England and Wales, does not exceed 14·6 per cent.

† Quêtelet, Sur l'Homme, &c. tome i. p. 258.

‡ This statement is contained in a letter from Prof. Jörg to Dr. M. Edwards, and is quoted by him in a paper, "De l'influence de la Température sur la Mortalité des Enfans nouveau-nés," in the Annales d'Hygiène Publique, t. ii. p. 304. For an account of the management of this institution, see E. G. Güntz, De viâ ac ratione quâ in Instituto Trieriano artis obstetriciæ usus et docetur et exercetur. Lipsiæ, 1837. 4to.

§ A Practical Treatise on Midwifery, by R. Collins, M.D. London, 1836. 8vo, pp. 500.

‖ It is a singular fact that trismus neonatorum is almost unknown in the hospitals of Paris. Some time since a friend mentioned to us that Professor Dubois stated in a letter to him, that in the whole course of his practice he had met with but one or two cases of this disease.

¶ Extracted in Froriep's Notizen, Band xix, No. xxi.

** Clinique des Maladies des Enfans noveau nés, Chap. ii. Paris, 1838. 8vo.

by any such cause, yielded, as we have seen, a precisely similar result. But surely these facts would not warrant any one in concluding that pneumonia attacks new-born infants in general in any such proportion.

It may perhaps be thought that similar causes cannot exert a pernicious influence upon the inmates of the Hôpital des Enfans Malades, who are not admitted below two years of age. All, however, who have been connected with that institution, concur in representing the mortality as very great. "It is necessary," says one of these gentlemen,* "to have frequented children's hospitals for any one to be able to form a correct notion of the injurious influence of an atmosphere loaded with the miasmata which are constantly disengaged from the bodies and clothes of the patients steeped in urine. It is doubtless to this circumstance that we must attribute the high rate of mortality in those wards which are occupied by the youngest children. If children but slightly indisposed are placed in one of these wards, they soon become weak and thin, and usually are seized by pneumonic catarrh or enterocolitis, diseases which under these circumstances are always very serious. The strongest constitution cannot shield them from the debility which results from the insalubrity of the air they constantly breathe, and this debility instead of preserving them from inflammations, seems to predispose certain organs to it."

But the following table† of the mortality in the Hôpital des Enfans Malades during the year 1822, will speak for itself:

| | Acute Diseases. | | | Chronic Diseases. | | | Convalescent | Total. |
|---|---|---|---|---|---|---|---|---|
| | Medical. | Variolous | Surgical. | Scrofula. | Tinea. | Itch. | | |
| Remaining on Jan. 1, 1822. | 75 | 13 | 64 | 79 | 83 | 30 | 9 | 407 |
| Entered during the year. | 1783 | 227 | 213 | 118 | 197 | 479 | 26 | 3142 |
| Died | 519 | 106 | 49 | 35 | 0 | 0 | 0 | 709 |
| Mortality. | 1 in 2 $\frac{41}{100}$ | 1 in 2 $\frac{15}{100}$ | 1 in 7 $\frac{10}{100}$ | 1 in 4 $\frac{33}{100}$ | 0 | 0 | 0 | 1in4$\frac{43}{100}$ |

The investigations of M. Becquerel‡ prove that a very large proportion of the deaths in this institution result from pneumonia. He says that between April and October, 1838, out of 421 patients labouring under acute diseases, dartrous or ophthalmic affections, 133 died, and on a post-mortem examination being made, the lungs of 49 were found to have been attacked by pneumonia. From this statement it results, that in more than one third of the fatal cases, death was produced, either directly or indirectly, by pneumonia, and this, too, at a season of the year when cold, which is regarded as one of the most powerful exciting causes of that disease, could have had but little influence in its production.§ From his observations M. Becquerel concludes that

* L. Senn, Recherches sur la Meningite Aigue des Enfans; Paris, 1825. 8vo, p. 125.
† Johnstone, Op. cit. p. 393.
‡ Archives Générales de Médecine, 1839, p. 437.
§ For many interesting details respecting the statistics of pneumonia, see Recherches Statistiques sur la Pneumonie, &c., par M. J. Pelletan, in the Bulletin de l'Académie Royale de Médecine, tome iv. p. 447.

pneumonia supervenes rarely in perfectly healthy children; that it occurs most frequently in those who are exhausted by previous disease, or placed in unfavorable hygienic conditions; and that it comes on in the course of acute diseases of specific adynamic character. He further regards uncomplicated pneumonia as rare, in which opinion he is confirmed by the testimony of all French writers, some of whom, as MM. Gerhard and Rufz, have even denied its existence as an idiopathic affection, while MM. Rilliet and Barthez, (p. 77,) though they do not deny its occasional occurrence, state that of forty cases of pneumonia in children under five years of age, only three were uncomplicated.

Thus much we thought it necessary to remark upon the peculiarities of the institutions in which the French have studied the diseases of children. But, having guarded our readers against hasty generalizations, we will now pass to the examination of the works whose titles stand at the head of this article. We have arranged them in what appeared to us to be the order of merit. MM. Rilliet and Barthez and M. Fauvel were all *internes* of the Hôpital des Enfans Malades, and the last-named gentleman is secretary to the Société Médicale d'Observation. All three are evidently careful and competent observers, and MM. Rilliet and Barthez possess the talent of saying much in a short compass, and clearly. M. Cruse, a physician at Königsberg, and one of the lecturers attached to the University, is a man of much learning and ingenuity, but not sufficiently careful to distinguish fact from theory, and much given to indulge in long and excessively tedious digressions. Sixty cases form the basis of the work of MM. Rilliet and Barthez; M. Fauvel builds a theory upon eight, the accuracy with which they were noticed making up, in his opinion, for the smallness of their number; while M. Cruse, who labours to establish the distinctions between pneumonia and bronchitis in children, does not mention the number of cases which came under his observation. We shall endeavour to give a condensed analysis of MM. Rilliet and Barthez' treatise, noticing as we proceed whatever may be particularly important in the remarks of M. Fauvel or of M. Cruse.

In selecting sixty observations for analysis, MM. Rilliet and Barthez have wisely left out of consideration all cases which came under their notice before they had prepared themselves for their task by a long and attentive study of the condition of the chest in healthy children. The results of their labours are arranged in tables at the end of the book, by which means the text is left unencumbered with statistical details, while many valuable facts may be ascertained at a single glance.

The first chapter treats, with much good sense, of the labours of their predecessors, but displays all that ignorance of what has been done in other countries, which is so characteristic of the French. M. Cruse, on the contrary, gives a very good sketch of the literature of the subject.

The second chapter discusses the morbid appearances left by inflammation of the lungs. We are here presented with the results of forty-three autopsies, to which if we add seven by M. Fauvel, we have a number whence we may hope to deduce something like accurate conclusions. The remarks with which MM. Rilliet and Barthez commence the chapter appear to us to furnish the clue to many of the disputes which have arisen concerning this disease. They observe (p. 15) that "in their anatomical investigations persons have not borne sufficiently in mind the connexion between the different forms of infantile pneumonia. They have studied each variety by itself, but have neglected to arrange them all under one head, and then to examine their mutual relations to each other." The plan which they have adopted has been first to describe each variety of pneumonia, next to examine those changes of the bronchi usually associated with it, and then to enumerate briefly the lesions of other organs which have been found to coexist with pneumonia.

The appearances left by vesicular pneumonia are especially deserving of note, since they may easily be confounded with the effects of phthisis, and were not referred to their true cause before the publication, in 1825, of a dissertation by M. Lanoix.* We do not recollect to have seen an account of this condition in any English work on diseases of children, and will therefore transcribe the very accurate description of it given by MM. Rilliet and Barthez.

"A lung thus affected is soft and flaccid, and collapses more or less according to the extent of the lesion. Its section presents a great number of granulations of about the size of a millet seed, and of a yellowish-gray colour. At a first glance these bodies might be taken for crude miliary tubercles, such as are often met with in the lungs of children; but a little attention shows that not to be the case, for these two species of granulations differ in their physical properties as much as in their nature. Tubercles form solid masses, pneumonic granulations contain a liquid. If a lung affected with tubercle is divided, some of the tubercles are cut by the scalpel and present their section on a level with the surface of the divided lung, while others slip from under the instrument and preserve their rounded shape. If these, too, are divided, they exhibit the appearance characteristic of tubercles. The granulations, on the other hand, collapse immediately on being divided, giving exit to a drop of puriform fluid, while those which are not cut by the knife still preserve their spherical form. If one of these granulations is opened with the point of a lancet, the puriform fluid escapes; but there remains in its centre a depressed point, which, however, is not always very easy to find. We once succeeded in tracing a very delicate canal some lines in length, with smooth parietes, and doubtless a small bronchial ramuscule, to its termination at this depressed point in the granulation. Their colour and general arrangement are, then, the only two characters which these morbid deposits have in common with each other." (pp. 17-18.)

MM. Rilliet and Barthez propose the term *vesicular bronchitis* as expressing the nature of this affection better than its usual name of *vesicular pneumonia*. They are of opinion that the disease is seated exclusively in the extreme bronchi; a certain number of pulmonary vesicles becoming inflamed, filled with pus, and dilated without the surrounding cellular tissue becoming implicated. To this explanation of the phenomenon, M. Fauvel (p. 68) objects, on the ground that inflammation of the pulmonary cells cannot be supposed to exist, without involving the surrounding cellular tissue. He imagines that one or more cells may be emptied of air by a forced expiration, and if this should be succeeded by a vigorous inspiration, the contents of the larger bronchi might be driven before the current of air which entered, and might thus reach the pulmonary cells. We confess that we are by no means satisfied with this hypothesis of M. Fauvel, and are the more inclined to subscribe to the opinion of MM. Rilliet and Barthez, from having found in a child who died of pneu-

* Essai sur la Pneumonie des Enfans comparée à celle des Vieillards. Théses de l'Ecole, 1825. No. cxix. p. 11.

monia some of these granulations in the upper part of the upper lobe of the right lung, although the bronchi of that lobe contained no unnatural secretion, and presented no sign of inflammation. If several neighbouring vesicles should be affected, the tissue connecting them may likewise inflame, and patches of *lobular pneumonia* would thus be formed.

Lobular pneumonia is an affection which we have reason to suppose to be more frequent in the Hôpital des Enfans Malades at Paris than among the children of the poor in London. The observations of Dr. Cuming, of Dublin,* would also lead us to suppose that it is unusual in the Irish metropolis. MM. Rilliet and Barthez recognize three degrees of it, corresponding to the three stages of ordinary pneumonia, and they propose further to distinguish the *pneumonie mammelonnée*, in which the inflamed lobules are distinctly circumscribed from the *pneumonie partielle*, in which there is no such definite line of demarcation between the healthy and the diseased structure. The *pneumonie mammelonnée* may pass on to suppuration and the formation of abscesses, the *pneumonie partielle* has a tendency to extend and involve an entire lobe. If the former has passed into the suppurative stage, a section of the lungs will present a number of cavities resembling in their form and disposition the appearance presented by the second degree of lobular pneumonia. These cavities sometimes communicate with the bronchi, though often that is not the case, and this latter circumstance, together with their irregular surface, may serve to distinguish them from the smooth and polished cavities formed by dilated bronchi. When lobular pneumonia has become general and has passed into the third stage, it is no longer distinguishable from ordinary lobar pneumonia, though at an earlier period their differences are obvious. In the latter the inflammation advances regularly from the part whence it set out, while in the former, patches of tissue in different stages of inflammation are scattered through the lungs. Pure lobar pneumonia is, according to their experience, not of very frequent occurrence; and when it proves fatal, the appearances left by it are similar to those produced by the disease in adults.

From their anatomical investigations, MM. Rilliet and Barthez conclude that all these lesions are only varieties of one disease, and that there is nothing special in any one of them; but that each is separated from the other only by a step, and one may become transformed into the other. The opinions of MM. Cruse and Fauvel differ widely from those just mentioned. We shall soon examine the facts collected by the latter gentleman.

*Carnification* of the lung is a condition far from uncommon, though it has not attracted the attention of any author with the exception of M. Rufz. The lung thus affected bears a considerable resemblance to the fœtal lung, and suggests the idea that the pulmonary cells have been obliterated by disease, and have lost their capacity for dilatation without continuing permanently engorged. This, they suggest, may be a form of chronic pneumonia; but they are not acquainted with any other condition of the lung to which the name of chronic pneumonia could with propriety be applied.

* On the Peripneumonia of Children; in vol. v. of Transactions of the King's and Queen's College of Physicians.

Tubercles were found only in fifteen out of forty-three cases, and by M. Fauvel in one of seven cases. These facts, which correspond with the results obtained by MM. Gerhard and Rufz, and which we can confirm from our own experience, present one of the most favorable features of pneumonia, since they show how rarely it is associated with one of the most frequent and most fatal complications of infantile disease.

The morbid conditions of the bronchi are especially note-worthy. In many instances their smaller ramifications present a considerably increased caliber, the dilatation sometimes affecting the tubes themselves, at other times merely their blind extremities. In two cases only were the parietes of the bronchial tubes thickened; in one of these instances they had acquired three times their natural thickness, a condition evidently chronic. This dilatation of the air-tubes was observed in all the seven cases recorded by M. Fauvel, who coincides with MM. Rilliet and Barthez in regarding it as the result of the accumulation of the secretions in the bronchi.

M. Fauvel agrees with his countrymen in representing the detection of inflammation of the bronchi as by no means an easy matter. They advise that shreds of the mucous membrane be torn off with the forceps and then examined, as the best means of avoiding error.

The nature of the bronchial secretions has been examined with great minuteness by M. Fauvel, (p. 38,) and it is upon the results thus obtained that he has founded his opinion of the identity in nature of croup and capillary bronchitis. On dividing the bronchi he usually found them full of a yellowish-white matter, containing little or no air, thick, and adherent to the mucous membrane. In many instances the air-tubes were found blocked up by this matter from their secondary divisions to their ultimate ramuscules. Where it was in contact with the wall of the bronchi it had the appearance of a false membrane, while in the interior it resembled pus. In four instances this pseudo-membranous appearance was well marked, existing on both sides of the chest and extending from the secondary bronchi to those of very small caliber. Now and then the false membrane could be traced into the most delicate ramifications, but usually it could not be followed beyond the smaller divisions of the bronchi. In a fifth subject the membrane was not well formed, being apparent only in patches; in a sixth the expectoration warranted the supposition that such matters had existed, though the patient did not die till three months afterwards and of another disease, when no concretions were found in the bronchi. In two other cases no false membranes were found, but only purulent matter. These cases are interesting, inasmuch as they exhibit a singular extent of disease of the bronchi, accompanied with very slight affection of the parenchyma of the lungs. They are not, nor indeed does M. Fauvel pretend that they are, altogether novel, analogous conditions having been described under the name of bronchial polypi by the medical writers of the last century. The appearance is alluded to, among others, by Michaelis,* and is treated of more fully by Dr. Badham,† who recognized the analogy between cynanche trachealis and acute bronchitis, and considered the latter to be merely an extension

* De Angina Polyposa, 8vo; sect. ii. Gottingæ, 1778.

† On the Inflammatory Affections of the Mucous Membrane of the Bronchiæ, 8vo, pp. 65, and in the Appendix; London, 1808.

of the disease which in croup is confined to the trachea. M. Fauvel quotes M. Jurine as having distinguished, under the name of *catarrhe suffocant aigu*, a species of croup which attacks especially the mucous membrane of the bronchi, and differs from true croup only in its seat.* It is to be regretted that M. Fauvel has confined his investigations to so small a number of cases as eight, instead of endeavouring, as he might have done, to ascertain the circumstances under which false membranes form in the air-passages, since we are much in the dark with reference to that interesting question.

Both MM. Rilliet and Barthez and M. Fauvel mention the existence of vesicular emphysema, but the last gentleman only seems to have met with interlobular emphysema, at which we are rather surprised, for our own experience would have led us to suppose that it is not such a very uncommon occurrence in young children.

The pleuræ were in many instances found inflamed; indeed they were perfectly healthy only in ten out of forty-three cases; and in a third of these the alterations they presented were quite recent.

We will not detain our readers with a minute account of the morbid conditions of the other organs, none of which were important except those met with in the intestines. The intestines could not be regarded as perfectly healthy in more than nine instances, but showed marks of disease usually of a chronic character, and consequently antecedent to the attack of pneumonia.

An account of the chief modifications of the respiratory sounds produced by inflammation of the lungs occupies the third chapter of the treatises of MM. Rilliet and Barthez, and of M. Fauvel. It must be obvious to every one that many circumstances concur to render the auscultation of the chest in young children a matter of great difficulty. Many persons indeed appear to have satisfied themselves that it is impracticable, and, like M. Berton, enumerate the obstacles which they have not perseverance to surmount. Even the careful investigations of M. Valleix have left so many points connected with the physical exploration of the chest in children undetermined, that we feel we are doing our readers good service in detailing somewhat at length the remarks of MM. Rilliet and Barthez.

In most cases of pneumonia, ronchus and sibilus may be heard at the commencement of the disease; but the irregularity of their seat, the shortness of their duration, and the very various sounds by which they are succeeded in different cases, render their diagnostic value very slight. In children above two years of age, however, these sounds become of greater importance, since in them pneumonia is more frequently consequent on bronchitis than at an earlier age.

The mucous ronchus is of more value than ronchus or sibilus, since it occurs oftener, persists for a longer time, and frequently coincides with bronchial respiration, while it is in other cases its immediate forerunner. These circumstances, too, impart to it an importance far beyond what we attach to it in the adult.

* M. Gluge, in his account of the influenza epidemic in Paris during 1837, mentions that in many cases false membranes, a quarter of a line in diameter, were found in the ramifications of the bronchi. Quoted by M. Cruse at p. 132.

Of all the râles, however, the sub-crepitant deserves the most attention, since it frequently precedes bronchial respiration, and is perceived in the very parts where bronchial respiration is subsequently heard. It is usually heard both in inspiration and expiration, though sometimes, when associated with bronchial respiration, it can be distinguished only during the former. In one instance it was detected only during expiration. Its duration was but short when it preceded bronchial respiration; when it followed it, however, it lasted for a much longer time, owing, as the writers observe, to the rapidity with which hepatization of the lungs supervenes in children and the slowness of its disappearance. We suspect that this extremely slow solution of hepatization is in a great measure attributable to the unfortunate hygienic condition of children in the Hôpital des Enfans Malades.

The existence of the *râle crépitant*, such as is heard in the pneumonia of the adult, has been denied by MM. Gerhard and Rufz, but it has been ascertained by MM. Rilliet and Barthez and also by M. Fauvel. It is heard only during inspiration, lasts but for a very short time, one or two days at the most, never reappears where it has once been heard, and is the immediate precursor of bronchial respiration. Being less frequent than the sub-crepitant râle, however, it may be regarded as of less value. A point of much importance in connexion with every variety of râle is the rapidity with which it disappears on placing the patient in the erect posture.

No modification of the respiratory sound is of such importance as bronchial respiration. It existed in two thirds of the cases, and when its existence was not ascertained, either the disease was very slight or it had become impossible to auscult the child during the last days of its life. In some instances the bronchial character was observed only during expiration, the inspiration remaining perfectly natural. This peculiarity was most remarkable in the youngest patients and at the commencement of bronchial respiration, or when it had begun to disappear. This phenomenon is regarded by MM. Rilliet and Barthez as indicating the existence of lobular pneumonia, the hepatized lobules playing the same part as crude tubercles do in a phthisical lung. In the youngest subjects, *râles* of various kinds preceded the bronchial respiration; in older patients an obscure respiration was often its precursor, while sometimes it appeared without having been preceded by any sign whatever. We invite our readers' attention to the following important caution:

"In most instances bronchial respiration is easily detected; but, nevertheless, the existence of certain *râles*, the impossibility of making children cough at pleasure, and their impatience during auscultation sometimes prevent it from being detected. But, not to dwell upon causes which interfere with our discovering this sound, we may enquire whether it can ever be confounded with other stethoscopic signs? Such an error is undoubtedly possible. In many instances we have convinced ourselves that persons but little skilled in auscultation, those especially who had but seldom explored the chest of children when in health, mistook exaggerated for bronchial respiration. But the difference between the two is in reality very considerable. How puerile soever respiration may be it always conveys the sensation of air entering vesicles; it is moreover audible only during inspiration, while bronchial respiration is heard principally during expiration." (p. 59.)

Percussion is a means of diagnosis much less valuable than auscultation. Not only does it not yield any result in lobular pneumonia, but, so great is the natural resonance of the chest in children, that a diminution of its sonoreity is often not appreciable, or at any rate can be detected only by comparison of the two sides of the chest with each other. In many instances, too, both lungs are affected, and we consequently lose this means of comparison. M. Hourmann attaches but little value to percussion, but thinks that the vibration of the voice, as perceptible to the hand, is greater on the diseased than on the healthy side.

In chapter iv. MM. Rilliet and Barthez compare the results of anatomical research with those afforded by auscultation; but we have not space to follow them through the interesting train of argument in which they endeavour to prove that the difference between the various forms of inflammation of the lungs of children is a difference of degree not of kind.

Chapter v. of MM. Rilliet and Barthez, and chapter ii. of M. Fauvel, are occupied with an examination of the causes of pneumonia. In most cases the patients were previously out of health; but MM. Gerhard and Rufz stated the fact too absolutely when they denied the existence of idiopathic pneumonia in children from two to five years of age. Idiopathic pneumonia continues, however, according to the experience of our authors, to be of rare occurrence up to the age of puberty. Age seems to exert a great influence in predisposing to pneumonia, and the disease is by far more frequent between two and five years of age than at any subsequent period. Forty of the sixty children whose cases form the basis of the work of MM. Rilliet and Barthez were under five years of age, and in 108 post-mortem examinations, M. Hache found pneumonia 71 times in children from two to five years of age, 37 times only in those from six to fifteen. But the age of the patient does not exert an influence merely in the production of the disease, it likewise modifies the form which it assumes. Some writers indeed have asserted that lobular pneumonia is peculiar to children between two and six years old; but to this observation, though true in the main, there are numerous exceptions.

Tables are introduced with the view of exhibiting the relative effect of season in predisposing to pneumonia, and also to show the diseases with which this affection is most usually combined in children; but the data are much too scanty to justify any positive conclusions. The following results, however, are worthy of commemoration: 1. Out of 94 cases of pneumonia, only 13 are recorded as simple idiopathic, the remaining 81 being complicated with other diseases. 2. Between the ages of two and five years measles is the disease most frequently complicated with pneumonia, and next in order chronic enteritis and hooping-cough. Between six and fifteen years of age measles stands first, then hooping-cough. It is added: "Though gangrene of the mouth occurs but thrice in this table, we may nevertheless venture to assert that of all the diseases of infancy this is the most frequently complicated with pneumonia, for we have met with it in every instance which has come under our notice, and M. Baudelocque assures us that this complication is invariably found to exist." (Rilliet and Barthez, p. 81.)

With regard to this invariable association of gangrene of the mouth with pneumonia, we will merely observe that not long since we examined the body of a child who had died from cancrum oris. The lungs were studded with tubercles, but presented no sign whatever of inflammation.

Dorsal decubitus and a prolonged residence in the hospital are two other causes of pneumonia to which our authors allude. After our remarks, however, at the commencement of this notice upon the hospitals of Paris, we will not again revert to the subject, but will notice one or two points of importance connected with the symptoms of pneumonia.

The condition of the pulse and respiration deserve especial notice, a sudden and rapid increase in their frequency often indicating the accession of pneumonia. In young children the pulse sometimes reached the rapidity of 150 or 180 beats in a minute, and usually ranged between 120 and 150. In children from six to fifteen years old it rarely attained so great a frequency as 140 or 150 pulsations in the minute, though it was usually 120 at the onset of the disease. In children between two and five years old the respiration varied from 30 to 80, in those from six to fifteen from 24 to 68 in the minute. We should have been inclined to mention a still higher figure as that of the occasional frequency of the inspirations in children from two to five years of age. In connexion with the frequency of the respiration, M. Fauvel mentions a fact, to which since we met with his treatise we have directed our attention, and which we believe to be correct. It is that the respiration does not continue to increase in frequency as the disease advances, but that it soon attains its greatest quickness, and afterwards shows slight diminution in its frequency, which is far from indicating any improvement. In illustration he subjoins a table from which it results, 1st, that the frequency of the respiration was always in inverse proportion to the age of the subject; 2d, that, except in one instance, its greatest frequency was observed at an earlier stage of the disease than its least frequency.

When pneumonia was uncomplicated, the frequency of the pulse and respiration was in proportion to the extent and severity of the inflammation, which circumstance likewise usually influenced the intensity of the febrile reaction. This statement, though quite in accordance with what we might expect to be the case, is yet at variance with the assertion of MM. Burnet and De la Berge, that lobular pneumonia is attended with a peculiar acceleration of pulse and respiration. MM. Rilliet and Barthez, however, remark that in all the cases detailed by those gentlemen, the condition of the pulse and respiration was sufficiently explained by the coexistence with the inflammation of the lungs of other diseases, as measles, typhus fever, &c.

We must refer our readers to chapters vii. and viii. for the able "*Tableau*" of the disease, and for the remarks on the diagnosis of pneumonia from pleurisy, bronchitis, and phthisis.

The prognosis (chapter ix.) is unfavorable in proportion to the youth of the patients; and that form of pneumonia which supervenes in the course of chronic affections is almost necessarily fatal. Secondary pneumonia is indeed at all ages a very dangerous disease, for of 81 cases which occurred under the care of the same physician in 1837, 77 terminated fatally. When pneumonia, however, comes on in healthy children or after a slight attack of catarrh, the patients usually do well, especially if they are above five years of age. To no one sign do MM. Rilliet and

Barthez attach so much importance as presaging an unfavorable termination as to the rapidity and especially the smallness of the pulse. With this character of the pulse there usually coincide cessation of the cough, coldness of the extremities, and a livid colour of the face, which serve to announce the speedy arrival of death.

The last chapter treats of the curative measures employed. These consisted of depletion, the administration of antimonials, and the application of rubefacients or blisters; but the writers observe that in almost all cases which terminated favorably, the first symptoms of improvement appeared about the same time, namely, from the seventh to the ninth day, whatever might have been the treatment, or even when no treatment was adopted. They attach but little value to the employment of depletion alone, but they speak more favorably of the administration of antimony, and observe that we need not fear to give tartar emetic in large doses, since tolerance is in general readily established, and there is but little reason to dread gastro-intestinal affections. They observe that tartar emetic appears to exert a more direct influence upon the pulse and the respiration than upon the hepatization of the lung, a statement to which we should feel disposed to add that when perfect tolerance of the medicine has been established, a perseverance in its use but seldom leads to any beneficial result; a combination of depletion with antimonials is the practice which they usually follow. The application of blisters they proscribe, and in this, as far as it regards children under three or four years of age, we most heartily coincide, for we cannot call to mind any instance in which the good they did was not more than counterbalanced by the troublesome and often dangerous sores they produced.

Eleven carefully reported observations and two tables, containing the results of the post-mortem examinations, conclude the treatise of MM. Rilliet and Barthez. We recommend it most earnestly to all our readers, and trust that it will be, as its authors promise, the first of many similar monographs on the diseases of children.

Dr. Küttner is physician to the Children's Dispensary at Dresden. His observations refer to forty cases of bronchitis or bronchio-pneumonia which came under his notice in the course of five years. In most points they confirm the statements of MM. Rilliet and Barthez, but do not present anything particularly novel. He notices the comparative immunity of children from the disease during the hottest months of the year, and the connexion of its prevalence with measles, influenza, and catarrhal affections in general. Two thirds of the cases occurred in children between the ninth month and the second year, but the disease occasionally appeared with all the peculiar features which it presents in infancy up to the fifth year. The detail of the symptoms presents nothing to remark, beyond the observation that in many of the cases, especially in those which terminated fatally, the children had been liable from birth to habitual shortness of breath, and to frequent attacks of catarrh.

More than half of the patients were in the enjoyment of robust health up to the time of their attack, but this more advantageous condition of the children at Dresden than of those in the hospital at Paris, was not attended with a proportionally low rate of mortality. Twenty-six of the forty children died, fourteen only recovered. All who were under the age of six months sank under the disease; the second year likewise was peculiarly fatal. The treatment consisted in leeching freely, and in the employment of calomel and tartar emetic; but many of the children were not brought to Dr. Küttner till after they had been neglected or maltreated for several days.

A post-mortem examination was made in seventeen cases. The bronchial mucous membrane was almost always red and injected, especially in the right lung, to which the inflammation was sometimes confined, and more rarely to the left, and the bronchial tubes contained more or less puriform mucus. The substance of the lung was inflamed in patches, principally at its posterior surface, and never pervading the whole of the organ. The pleura was inflamed and adherent to the ribs in seven cases, and in one instance sero-purulent effusion had taken place into the pericardium, and the surface of the heart was coated with a false membrane.

---

